# Tau accumulation impairs mitophagy *via* increasing mitochondrial membrane potential and reducing mitochondrial Parkin

**DOI:** 10.18632/oncotarget.7861

**Published:** 2016-03-02

**Authors:** Yu Hu, Xia-Chun Li, Zhi-hao Wang, Yu Luo, Xiangnan Zhang, Xiu-Ping Liu, Qiong Feng, Qun Wang, Zhenyu Yue, Zhong Chen, Keqiang Ye, Jian-Zhi Wang, Gong-Ping Liu

**Affiliations:** ^1^ Department of Pathophysiology, School of Basic Medicine and The Collaborative Innovation Center for Brain Science, Key Laboratory of Ministry of Education of China for Neurological Disorders, Tongji Medical College, Huazhong University of Science and Technology, Wuhan, China; ^2^ Department of Pharmacology, College of Pharmaceutical Sciences, Zhejiang University, Hangzhou, China; ^3^ Departments of Neurology and Neuroscience, Friedman Brain Institute, Icahn School of Medicine at Mount Sinai, New York, NY, USA; ^4^ Department of Pathology and Laboratory Medicine, Emory University School of Medicine, Atlanta, GA, USA; ^5^ Co-innovation Center of Neuroregeneration, Nantong University, Nantong, JS, China

**Keywords:** tau, mitophagy, PINK1, Parkin, Alzheimer's disease, Gerotarget

## Abstract

Intracellular accumulation of wild type tau is a hallmark of sporadic Alzheimer's disease (AD). However, the molecular mechanisms underlying tau toxicity is not fully understood. Here, we detected mitophagy deficits evidenced by the increased levels of mitophagy markers, including COX IV, TOMM20, and the ratio of mtDNA to genomic DNA indexed as mt-Atp6/Rpl13, in the AD brains and in the human wild type full-length tau (htau) transgenic mice. More interestingly, the mitophagy deficit was only shown in the AD patients who had an increased total tau level. Further studies demonstrated that overexpression of htau induced mitophagy deficits in HEK293 cells, the primary hippocampal neurons and in the brains of C57 mice. Upon overexpression of htau, the mitochondrial membrane potential was increased and the levels of PTEN-induced kinase 1 (PINK1) and Parkin decreased in the mitochondrial fraction, while upregulation of Parkin attenuated the htau-induced mitophagy deficits. Finally, we detected a dose-dependent allocation of tau proteins into the mitochondrial outer membrane fraction along with its cytoplasmic accumulation. These data suggest that intracellular accumulation of htau induces mitophagy deficits by direct inserting into the mitochondrial membrane and thus increasing the membrane potential, which impairs the mitochondrial residence of PINK1/Parkin. Our findings reveal a novel mechanism underlying the htau-induced neuronal toxicities in AD and other tauopathies.

## INTRODUCTION

Mitochondria play crucial roles in energy production, the synthesis of key metabolites, apoptosis regulation, calcium buffering, and generation of endogenous reactive oxygen species [1, 2]. Since the neuronal activities, such as synaptic transmission, axonal transport, dendritic transport, ion channels and ion pump activity, are extremely energy-dependent [3], neurons are particularly sensitive to the changes of mitochondrial functions. Therefore, mitochondrial damage has been reported to be associated with many neurodegenerative diseases, including Alzheimer's disease (AD), Parkinson disease, and Huntington's disease [4-6].

The control of mitochondrial mass is through mitochondrial biogenesis and degradation. PINK1/parkin-dependent mitophagy contributes to mitochondrial quality control by selectively eliminating the dysfunctional mitochondria and maintain mitochondrial integrity and function [7]. PINK1 and the E3 ubiquitin-protein ligase parkin are two important protein mediators in the process of mitophagy. When the membrane potential of a mitochondrial segment depolarized, PINK1 accumulates on the outer mitochondrial membrane, then its kinase activity recruits parkin to the mitochondria [8, 9]. After mitochondrial residing, the parkin-mediated ubiquitination of mitochondrial substrates, such as voltage-dependent anion-selective channel protein (VDAC1) and mitofusins (MFN1/2), will lead to the recruitment of p62/SQSTRM and LC3 [10-12]. Finally, the mitochondrion whose membrane potential dissipated is engulfed into the autophagosome and eliminated through mitophagy.

Abnormal accumulation of wild type tau proteins is a hallmark of sporadic AD [13]. Expression of full-length human tau alone causes intracellular tau pathologies and behavioral deficits in mice [14, 15], while turning off htau expression attenuates the pathologies [16]. Reduction of endogenous tau also ameliorates memory deficits caused by β-amyloid (Aβ) [17]. However, how intracellular accumulation of the wild type tau impairs cells' function and eventually leads to neurodegeneration is currently not fully understood.

Mitochondrial dysfunction is an early pathological event of AD [18, 19], and abnormal mitochondrial morphology and distribution are detected in the postmortem AD brains and their fibroblasts [4, 20]. Recent studies suggest that there is an intrinsic link between tau and mitochondria. For instance, an N-terminal truncated tau (20-22kDa) is largely enriched in mitochondria of the AD brains and its amount in nerve terminal fields correlates with the pathological synaptic changes [21]. Mitochondrial dysfunction is also detected in P301L tau transgenic mice [22]. Deregulation of mitochondrial complex I with aging is tau dependent [23]. Tau phosphorylation can antagonize cell apoptosis with the mechanisms involving Bcl-2 and caspase-3 in mitochondria [24-26], while expression of fusion proteins attenuates apoptosis [27]. These observations suggest that intracellular accumulation of tau may cause neurodegeneration through disrupting mitochondrial functions, but the direct evidence for the role of wild type full-length tau in the mitophagy is still lacking.

In the present study, we show that intracellular accumulation of human wild type full-length tau, as seen in the sporadic AD brains, induces mitophagy deficits, with the mechanisms involving a direct insertion of htau into the outer membrane fractions of mitochondria, an increased mitochondrial membrane potential and an impaired mitochondrial residence of PINK1/Parkin.

## RESULTS

### Mitophagy deficit is associated with abnormal tau accumulation in the brains of AD patients and the AD-like htau transgenic mice

**Figure 1:**
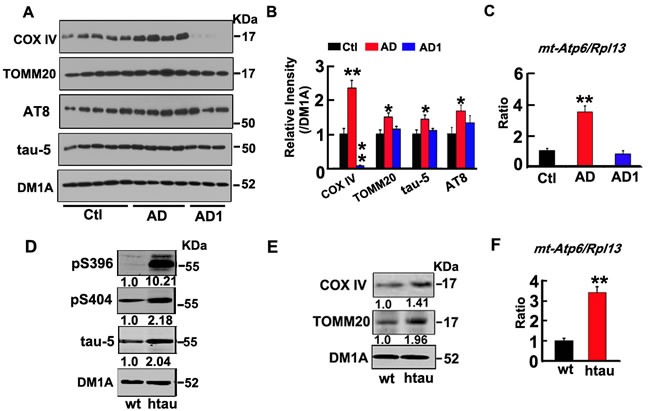
Tau-correlated mitophagy deficits were found in the brains of AD patients and the htau transgenic mice **A.**, **B.** Compared with the age-matched non-AD controls, the levels of mitochondrial marker proteins (COX IV and TOMM20), total tau proteins (tau-5) and phosphorylated tau (AT8) were increased in the homogenates of AD brains measured by Western blotting. DM1A against tubulin was used as a loading control. **C.** The increased ratio of mt-Atp6/Rpl13 (mitochondria-encoded DNA/nucleus-encoded DNA) measured by real-time PCR. **D.** The increased total tau (tau-5), and phosphorylated tau (pS396, pS404), and **E.** mitochondrial marker proteins (COX IV and TOMM20) in htau transgenic mice (htau) compared with the age-matched wild type littermates (wt) measured by Western blotting. **F.** The increased ratio of mt-Atp6/Rpl13 in htau mice measured by real-time PCR. Data were expressed as mean±SD. *, *p* < 0.05, **, *p* < 0.01 *vs* Ctl (the control) or wt (age-matched wild type littermates).

To explore whether mitophagy deficit is involved in AD pathogenesis, we analyzed the levels of mitophagy markers, including COX IV, TOMM20, and the ratio of mtDNA to genomic DNA, indexed as mt-Atp6/Rpl13 by Western blotting or real-time PCR. We found that levels of COX IV, TOMM20 and the ratio of mt-Atp6/Rpl13 were all significantly increased in the homogenates of AD brains (Figure 1A-1C), indicating increases of mitochondrial number. Interestingly, we observed that the mitophagy deficit seemed correlated with abnormal tau accumulation, i.e., the increase of COX IV and TOMM20 was only shown in those AD patients who had high level of tau proteins (AD group), not AD patients (AD1 group) whose total tau had no significant change compared with the nondemented control (Figure 1A-1C). The mitophagy deficits were also detected in the hippocampus of 6m-old htau transgenic mice (STOCK Mapttm1(EGFP)Klt Tg(MAPT)8cPdav/J) when compared with the age-matched littermates (Figure 1E, 1F), with significant tau accumulation (Figure 1D). The perinuclear mitochondrial accumulations were also shown by electron microscopy (unpublished data). These data suggest an intrinsic association of intracellular tau accumulation with mitophagy deficits.

### Overexpression of htau induces mitophagy deficits both *in vitro* and *in vivo*

**Figure 2:**
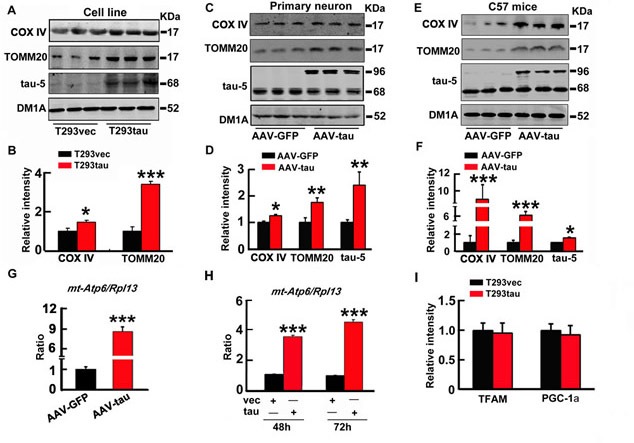
Overexpression of htau induces mitophagy deficits *in vitro* and *in vivo* **A.**, **B.** The human wild type full-length tau (htau) plasmid was transiently expressed in HEK293 cells for 48 h (T293tau), **C.**, **D.** EGFP-labeled AAV-htau was overexpressed in primary hippocampus neurons (7 *div*) for 48 h, or **E.**, **F.** The eGFP-labeled AAV-htau was stereotaxically infused into the hippocampal CA3 of 3m-old C57 mice for 1 month, and then, levels of mitochondrial marker proteins COX IV and TOMM20, and total tau (tau-5) in the cell or brain lysates were detected by Western blotting. **G.**, **H.** The ratio of mt-Atp6/Rpl13 in the hippocampus of c57 mice (G) or HEK293 cells at 48 h or 72 h after serum deprivation (H) measured by real-time PCR. **I.** Human wild type full-length tau (T293tau) or the vector (T293vec) plasmid was transiently expressed in HEK293 cells for 48 h, then the mRNA levels of PGC-1α (peroxisome proliferator-activated receptor γ coactivator 1α) and TFAM (mitochondrial transcription factor A) were measured by real-time PCR. Each experiment was repeated for at least 3 times, and data were expressed as mean±SD. *, *p* < 0.05; **, *p* < 0.01; ***, *p* < 0.001 *vs* T293vec or AAV-GFP.

To verify the role of tau in mitophagy deficits, we overexpressed htau plasmid in HEK293 cells for 48 h (termed T293tau, Figure 2A, 2B), or the eGFP-labeled AAV2/8-htau in primary hippocampal neurons (7 *div*) for 48 h (Figure 2C, 2D), or in the hippocampal CA3 region of 3m-old c57 mice for 1 month by stereotaxic injection (Figure 2E, 2F), and the image of AAV2/8-eGFP transfected hippocampal CA3 region was shown as Supplementary Figure 1. Then, the levels of mitophagy markers together with tau proteins were measured by Western blotting. Compared with the vector transfected controls, the levels of COX IV, TOMM20 (Figure 2A-2F) and the ratio of mt-Atp6/Rpl13 (Figure 2G, 2H) were increased by overexpression of htau both *in vitro* and *in vivo*. We also found that the autophagy proteins Lc-3 II and p62 significantly increased (Supplementary Figure 2). Furthermore, a time-dependent increase of mt-Atp6/Rpl13 (~3.4-fold and 4.5-fold respectively at 48 h and 72 h) was detected in T293tau cells compared to the vector-transfected controls (T293vec) (Figure 2H). To confirm whether the increase of mitochondria is due to the mitophagy deficit or mitochondrial biogenesis, we detected mRNA levels of peroxisome proliferator-activated receptor γ coactivator 1α (PGC-1α) and mitochondrial transcription factor A (TFAM), two key transcription factors involved in the mitochondrial biogenesis [28, 29]. The results showed that overexpression of htau did not change the mRNA levels of PGC-1a and TFAM (Figure 2I). These are direct evidence showing that the htau accumulation blocks mitophagy.

### Overexpression of htau increases mitochondrial membrane potential with mitophagy deficits and a decreased mitochondrial residence of PINK1/Parkin

**Figure 3:**
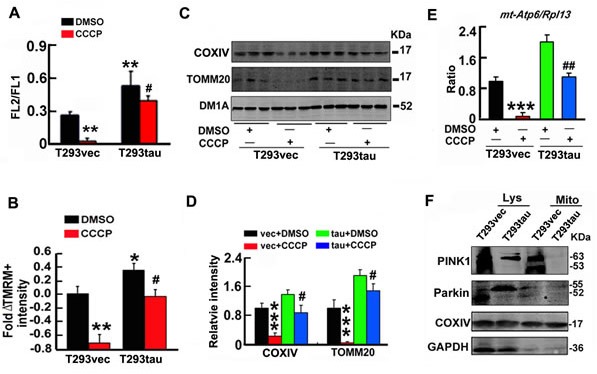
Tau increases mitochondrial membrane potential and induces mitofusin accumulation **A.** T293tau or T293vec cells were treated with CCCP (20 μM) or DMSO (vehicle control) for 30 min, then the membrane potential (FL_2_/FL_1_) was measured by JC-1 staining (*n* = 10). **B.**T293tau or T293vec cells were incubated with TMRM (20 nM) for 30 min, and then the fold ΔTMRM^+^ intensity (ΔΨm) was analyzed. **C.**-**E.** Levels of mitochondrial marker proteins (COX IV and TOMM20) and ratio of mt-Atp6/Rpl13 were detected by Western blotting (C, D) and real-time PCR (E), respectively, in T293tau and T293vec cells after treatment with CCCP (20 μM for 30 min). (F) Levels of PINK1 and Parkin in cell lysates (Lys) and mitochondria (Mito) fractions measured by Western blotting. Data were expressed as mean±SD. *, *p* < 0.05; **, *p* < 0.01, ***, *p* < 0.001 *vs* T293vec+DMSO; #, *p* < 0.05, ##, *p* < 0.01 *vs* T293tau+DMSO.

The membrane potential is critical for the mitochondrial residence of PINK1/Parkin and the subsequent ubiquitination-associated mitophagy [7-12]. Therefore, we measured the mitochondrial membrane potential. Unexpectedly, we observed a significant increase of mitochondrial membrane potential in the cells with overexpression of htau (Figure 3A). To confirm the influence of htau on mitochondrial membrane potential, we used carbonyl cyanide m-chlorophenylhydrazone (CCCP), a mitochondrial membrane depolarizer that can decrease mitochondrial membrane potential and thus causes proteolysis of the mitochondrial proteins by mitophagy [30]. We observed that CCCP treatment remarkably decreased the membrane potential in control cells, but only a slight reduction was shown in the htau-expressing cells measured by JC-1 or tetramethyl rhodamine methyl ester (TMRM) (Figure 3A, 3B). We also found that levels of COX IV and TOMM20 were significantly decreased, while LC-3 II significantly increased after CCCP treatment in the control cells, whereas only a slight reduction of COX IV and TOMM20 was seen in the htau-expressing cells (Figure 3C, 3D, and Supplementary Figure 3). Similarly, CCCP treatment reduced mtDNA in both control and htau transfected cells, but the reduction in the tau-expressing cells was much less than the control cells (Figure 3E). The mitochondrial membrane potential affects the residence of PINK1 and Parkin in mitochondria [7-12]. With an increased mitochondrial membrane potential by htau accumulation, we observed a diminished PINK1 and a significantly decreased level of Parkin in the mitochondrial fraction (Figure 3F). These data together suggest that htau accumulation may induce mitophagy deficits by increasing mitochondrial membrane potential and disrupting the mitochondrial residence of PINK1/Parkin.

### Upregulating parkin rescues the htau-induced mitophagy deficits

**Figure 4:**
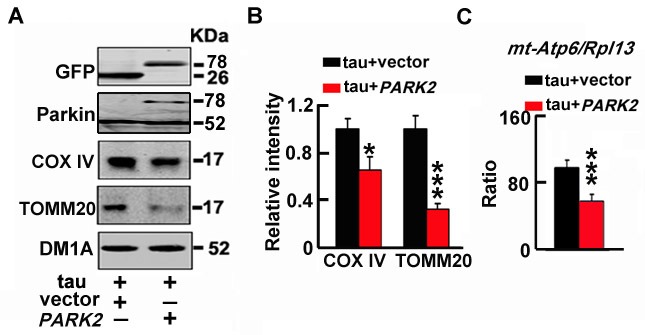
Up-regulating parkin rescues the htau-induced mitophagy deficits **A.**, **B.** T293tau cells were transfected with wild type *parkin* (PARK2) plasmid for 48 h, and then the mitochondrial marker proteins (COX IV and TOMM20) were measured by Western blotting. **C.** The ratio of mt-Atp6 and Rpl13 was assessed by real time PCR. Each experiment was repeated at least 3 times. The data are expressed as mean±SD. *, *p* < 0.05, ***, *p* < 0.001 *vs* vec.

To validate the role of the reduced Parkin in htau-induced mitophagy deficits, we overexpressed wild-type parkin (*PARK2*) in T293tau cells. The results showed that simultaneous upregulation of Parkin attenuated the htau-induced mitophagy impairment (Figure 4A-4C), which confirm the role of Parkin reduction in the htau-induced mitophagic deficits. We also found that total tau and phosphorylated tau levels had no significant change while parkin overexpression (Supplementary Figure 4).

### Overexpression of htau increases tau levels in mitochondrial outer membrane fraction

**Figure 5:**
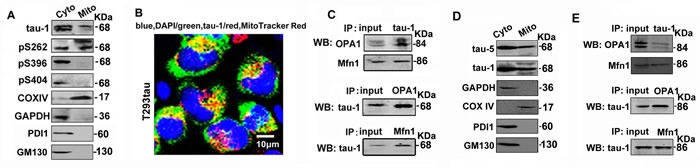
A direct interaction of tau with mitochondrial marker proteins **A.** The cytoplasmic (Cyto) and mitochondrial (Mito) fractions were prepared from T293tau cells, and tau was analyzed by using a panel of phosphorylation site-specific antibodies, including tau-1 (reacts with the unphosphorylated tau at Ser198/199/202), pS262, pS396 and pS404. **B.** The representative image shows co-staining of tau-1 (green) with MitoTracker Red in T293tau cells. **C.** Co-immunoprecipitation data show association of tau with mitochondrial marker proteins OPA1 and Mfn1. **D.** The cytoplasmic (Cyto) and mitochondrial (Mito) fractions were prepared from the hippocampus of htau transgenic mice, and tau was analyzed by using anti-tau-1 antibody. **E.** Co-immunoprecipitation data show association of tau with mitochondrial marker proteins OPA1 and Mfn1 in hippocampal extracts of htau transgenic mice. GAPDH is a marker of cytoplasmic proteins, while TOMM40 and COXIV are respectively markers of mitochondrial outer membrane, inner membrane and intermembrane space proteins. PDI or GM130 is the marker of ER or Golgi apparatus used to prove the purity of the mitochondrial fraction.

To further explore whether tau directly interacts with mitochondria, we first measured the level of tau in the mitochondrial fraction after transient expression of tau in HEK293 cells. Interestingly, the total tau (tau-5), unphosphorylated tau at tau-1 epitope and the phosphorylated tau at Ser262 (located at the microtubule binding domain of tau) were detected in the mitochondrial fraction, while the C-terminal Ser396- and Ser404-phosphorylated tau were not detected (Figure 5A). Immunofluorescence staining data show co-localization of tau-1 epitope tau with MitoTracker Red in the elongated mitochondria (Figure 5B). Furthermore, N-terminal tau-1 epitope tau was co-immunoprecipitated with mitochondrial protein OPA1 and Mfn1 (Figure 5C). We confirmed the above results in htau transgenic mice by showing tau-1- and tau-5-postive tau proteins in the mitochondrial fraction (Figure 5D), and tau-1 co-immunoprecipitation with OPA1 and Mfn1 (Figure 5E). These data provide strong evidence for a direct interaction of tau with mitochondria.

**Figure 6:**
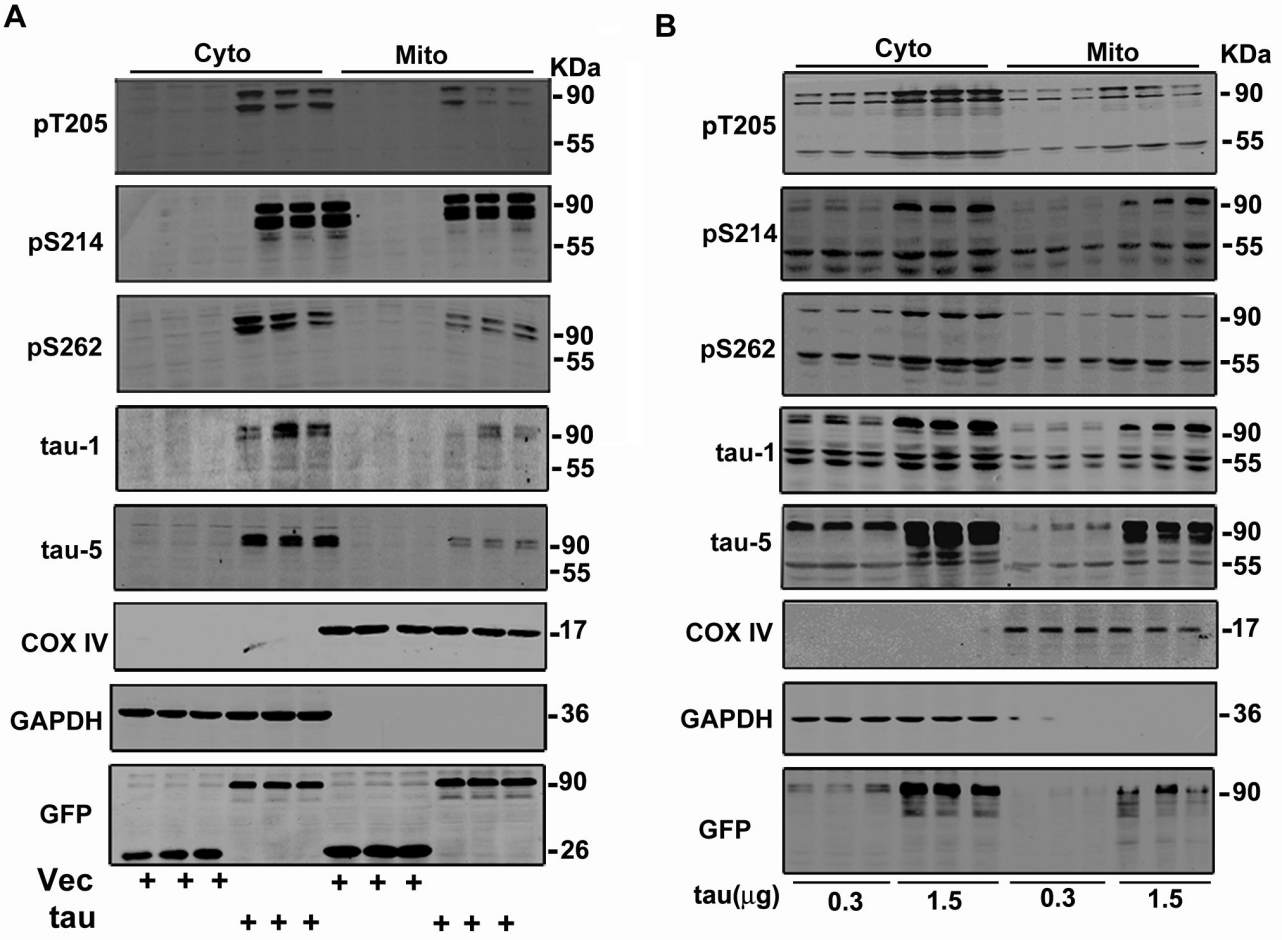
A dose-dependent allocation of tau into the mitochondria accompany with the intracellular tau accumulation **A.** Cytoplasmic expression of htau in a N2a cell line that only expresses negligible endogenous tau protein causes mitochondrial location of tau proteins. **B.** Cytoplasmic overexpression of different concentrations of htau in a N2a cell line that expresses low level of endogenous tau protein causes a dose-dependent elevation of mitochondrial tau protein.

To identify the relationship of mitochondrial location of tau with its cytoplasmic accumulation in neural cells, we first transiently expressed htau in a selected Neuro 2a (N2a) cell line, which expresses negligible endogenous tau protein (Figure 6A). With the cytoplasmic tau accumulation, apparent tau signals were detected in the mitochondrial fraction (Figure 6A). To better mimic the AD-like intracellular tau accumulation, we transfected two different dosages of htau plasmid into another N2a cell line that expresses endogenous tau proteins [24]. Along with a dose-dependent cytosolic accumulation of tau, exogenous overexpression of htau increased the mitochondrial levels of total tau (tau-5), phosphorylated tau at pT205, pS214, and pS262, and the unphosphorylated tau at tau-1 epitope with major molecular mass of ~90 KDa and ~55 KDa (Figure 6B). These data confirm that intracellular tau accumulation can be dose-dependently allocated into the mitochondria.

**Figure 7:**
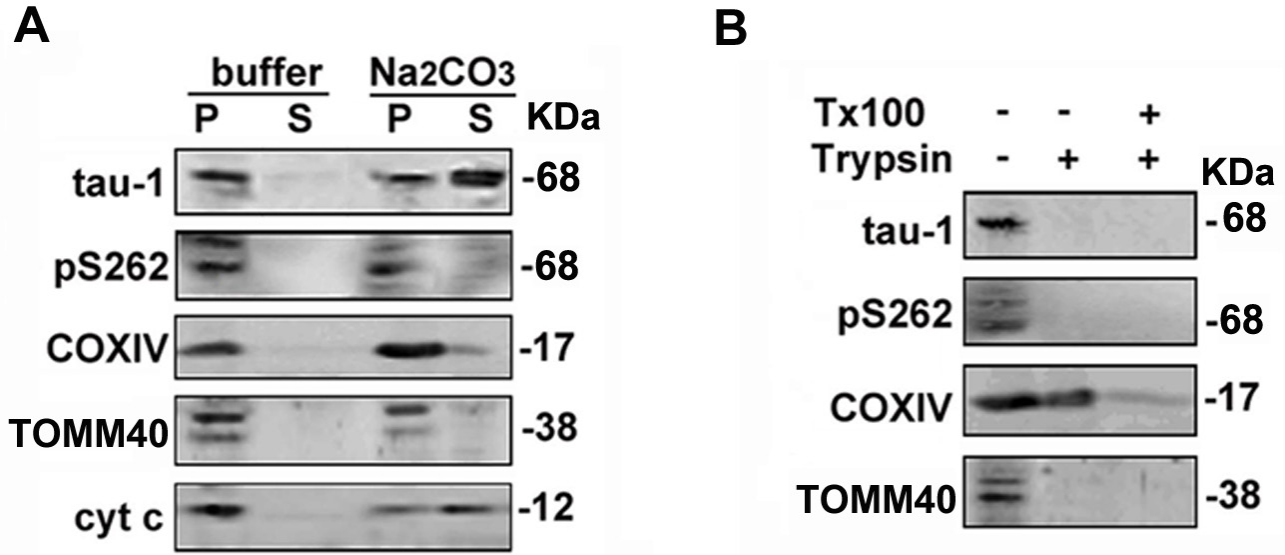
Tau may span on the mitochondrial outer membrane with some domains exposing to the inter-membrane spaces **A.** The mitochondrial fractions prepared from T293tau cells were re-suspended in buffers with or without Na_2_CO_3_ (0.1 mol/L, pH 11.5) followed by centrifugation, and the supernatants (S) or membrane pellets (P) were detected with the indicated antibodies by Western blotting. **B.**Mitochondrial fractions were digested with trypsin in the absence or presence of 1% Triton X-100 (Tx-100) for 30 min or with mock control, and the protein level of tau was analyzed. TOMM40, COXIV and cyt c are respectively markers of mitochondrial outer membrane, inner membrane and intermembrane space proteins.

To validate the topology of tau in mitochondria, we treated the purified mitochondria with a high-pH wash (Na_2_CO_3_). Like cytochrome c (cyt c), a protein marker of mitochondrial intermembrane space, the majority of tau-1 reactive tau was dissolved into the supernatant fraction after Na_2_CO_3_ treatment, suggesting the mitochondrial intermembrane space location of the majority unphosphorylated tau (Figure 7A). Trypsin can digest mitochondrial outer membrane proteins, such as TOMM40, while trypsin plus Triton-X100 can remove the mitochondrial inner membrane proteins, such as COX IV [31]. We found that treating the purified mitochondria with trypsin alone completely removed tau proteins, as seen in TOMM40 (Figure 7B). These data strongly suggest that tau may span on the mitochondrial outer membrane with some domains exposing to the inter-membrane spaces.

## DISCUSSION

Intracellular accumulation of wild type tau is the major cause of neurodegeneration in sporadic AD; however, the mechanism is not fully elucidated. Tau aggregation is associated with mitochondrial damage, oxidative stress as well as structural and functional alterations of neurons in AD [32-34]. As efficient mitochondria clearance by mitophagy is a key mechanism for mitochondrial quality control, here, we investigated the effects of tau on mitophagy and the molecular mechanisms. We find that intracellular accumulation of human wild type full-length tau, as seen in sporadic AD brains, results in mitophagy deficits. Although mitochondrial abnormality has been observed in AD patients and in transgenic mice expressing P301L mutated tau (the latter is only seen in patients of FTDP-17 but not AD), and the N-terminal truncated tau proteins were detected in mitochondria [18-23], our current study provides the first direct evidence showing that intracellular accumulation of wild type full-length tau inhibits mitophagy, which reveal a novel mechanism underlying tau-induced neurodegeneration in sporadic AD patients.

The mitophagy deficits can be evaluated by accumulation of mitochondrial marker proteins and the increase of mtDNA [35]. In neurons, autophagosomes actively form along neurites and in synapses but efficient clearance of the autophagic vesicles requires their retrograde transport towards the neuronal cell body, where the lysosomes are enriched. Microtubules serve as tracks for the intracellular transportations and tau accumulation blocks the transport. We speculate that the increased htau may block transport of the autophagosomes, thus lead to a massive accumulation of the impaired mitochondria into somatodendritic compartment of the cells, which is consistent with the reports that damaged mitochondria with altered morphology per neuronal perikarya were significantly increased in AD brains [36]. Mitochondrial fusion and fission also play a crucial role in mitophagy, in which, fission has been considered as a prerequisite for mitophagy [37, 38], because autophagy cargos with a large size cannot be efficiently engulfed by phagophores [7, 39-41]. A previous study in drosophila and fibroblast showed that overexpression of human R406W mutant tau could elongate mitochondria with the mechanisms involving a reduced fission [42]. The effect of wild type full-length tau accumulation on mitochondrial fusion or fission deserves further investigation.

The PINK1/Parkin signaling is critical for degradation of the mitochondrial fusion proteins and a selective mitophagy processing [7-12]. Parkin is responsible for ubiquitinating substrates on the mitochondrial outer membrane and subsequent recruitment of autophagy machinery [8-10]. We found that reduced PINK1 and Parkin in the mitochondrial fraction of the htau-transfected cells. Although the mechanisms underlying PINK1 recruiting Parkin to the mitochondria are not fully understood, Mfn2 phosphorylation by PINK1 was considered as a key step [43]. The important role of Parkin deficits in htau-induced mitochondrial impairments was further demonstrated by up-regulating Parkin, which rescued the htau-induced mitophagy deficits. Previous studies reported that overexpression of Parkin decreased intracellular Aβ levels and extracellular plaque deposition, attenuated caspase activity, prevented mitochondrial dysfunction and oxidative stress and restored neurotransmitter synthesis [36, 44]. Here, we show that overexpression of Parkin attenuates the htau-induced mitophagy deficits, suggesting that parkin may serve as a potential target for AD therapy.

A previous study showed that overproduction of β-amyloid reduced membrane potential [45]. It was also reported that the triple transgenic AD mice or the aged P301L tau transgenic mice exhibited mitochondrial dysfunction but with no changes on mitochondria membrane potential [23, 24]. In the current study, we observed unexpectedly a significantly increased mitochondrial membrane potential upon overexpression of htau. Furthermore, treatment with CCCP significantly reduced the membrane potential in control cells but not to the same levels in htau expressing cells. Low membrane potential is required for the mitochondrial residency of PINK1/Parkin [30], therefore, the increased membrane potential may underlie the htau-induced reduction of PINK1/Parkin in mitochondria. Since the mitochondrial tau level was significantly increased accompanying with a dose-dependent overexpression of htau, we speculate that tau may increase the membrane potential by direct insertion into the outer membrane fraction. Interestingly, we only detected N-terminal tau but not C-terminal phospho-tau in the mitochondria. A most recent study detects N-terminal tau in the mitochondrial membrane fraction [46], which supports our findings of the increased membrane potential by the mitochondrial tau insertion. Mitochondrial abnormality is undoubtedly associated with the pathogenesis of AD. There are some contradictory studies about the levels of mitochondrial proteins and mtDNA in the AD brains. It was shown that COX activity in post mortem AD brains was decreased [47, 48]. Moreover, the reduced cellular expression of COX subunit II and IV more pronounced in AD [49]. Hirai et al also found that some neurons showing increased oxidative damage have a striking and significant decrease in mtDNA and cytochrome oxidase in the brain of AD patients. However, they also found that pyramidal neurons show increased mtDNA and cytochrome oxidase in the hippocampus of AD [50]. In the present study, we found that tau accumulation induced mtDNA and mitochondrial proteins increased. Why the mitochondrial study in the AD had these contradictory reports, we speculated that, though Aβ and tau aggregation are associated with mitochondrial damage, oxidative stress as well as structural and functional alterations of neurons in AD [32-34], it seems that Aβ and tau proteins induces mitochondrial dysfunction and mitochondrial number with different mechanisms, which waiting for further study.

In non-apoptotic cells, JC-1 shows green color in cytoplasm (maximal excitation 510 nm, maximal emission 527 nm) and it turns red in mitochondria (maximal excitation 585 nm, maximal emission 590 nm). Treatment with 10 μM CCCP for 20 min can significantly reduce the red/green ratio, suggesting decrease of membrane potential [51]. However, we did not see significant change of JC-1 staining in htau-expressing cells by using 10 μM CCCP for 20 min, thus we used 20 μM CCCP for 30 min. Mitochondrial fusion occurred in daughter-mitochondria that show high membrane potential [52]. We speculate that expression of htau may increase mitochondrial fusion, which may explain why we do not see significant reduction of the membrane potential in htau-expressing cells when low concentration of CCCP was used.

Taken together, we find in the present study that intracellular accumulation of human wild type full-length tau, as seen in sporadic AD brains, induces mitophagy deficits with the mechanisms involving in causal-correspondently an increased mitochondrial membrane potential and an impeded mitochondrial residency of PINK1/Parkin.

## MATERIALS AND METHODS

### Plasmids, antibodies and reagents

Human pIRES-eGFP-Tau40 plasmid was a gift of Dr. Khalid Iqbal (New York State Institute for Basic Research in Developmental Disabilities, Staten Island, NY). The PCI-neo-Tau40 was constructed in our lab. For GFP-*parkin* (PK2, the full-length mouse *parkin* cDNA) was cloned into pEFGP-C1 plasmid (Clontech). EGFP-labeled AAV2/8-htau and its control virus were purchased from OBio Biologic Technology Co., Ltd.

Mouse monoclonal antibody (mAb) anti-OPA1 and anti-GM130 were from BD Bioscience; mAb anti-Mfn1 was from Santa Cruz; mAb anti-COXIV, anti-GAPDH, anti-Cytc, anti-GFP, anti-PDI1 and pAb anti-ubiquitin, anti-PINK1, anti-Parkin, anti-Mfn2, anti-SQSTM/p62, anti-LC and anti-TOMM40 were from Abcam; mAb anti-α-tubulin was from Sigma; two kinds of mAb anti-tau-5 were from NeoMarkers or Millopore, mAb tau-1 was from Chemicon, pAbs against phosphorylated tau (pT205, pS214, pS262, pS396 or pS404) were from BioSource; mAb AT8 (tau phosphorylated at Ser202 and Thr205) was from Thermo Fisher Scientific, pAb anti-TOMM20 was from Anbo Biotechnology, CCCP/JC-1 was from Sigma; Lipofectamine2000 and TMRM was from Invitrogen.

### Cell culture

The human embryonic kidney293 (HEK293) were grown in Dulbecco's Modified Eagle's medium (DMEM medium) (12491-015, Gibco), supplemented with 10% (v/v) fetal bovine serum and 1% penicillin/streptomycin, in a humid 5% CO_2_ incubator at 37°C. After growing 24 h in plates or flasks, the cells were transfected with the indicated plasmid(s) using Lipofectamine2000 according to the manufacturer's instructions.

For primary neuron cultures, 18 days embryonic (E18) rat hippocampus were seeded at 30,000-40,000 cells per well on 6-well plates coated with Poly-D-Lysine/Laminin (Bioscience) in neurobasal medium (Invitrogen) supplemented with 2% B27/0.5 mM glutamine/25 mM glutamate. Half the culture medium was changed every 3 days with neurobasal medium supplemented with 2% B27 and 0.5 mM glutamine. All cultures were kept at 37°C in a humidified 5% CO_2_ containing atmosphere. More than 90% of the cells were neurons after they were cultured for 7 to 17 *div*; this was verified by positive staining for the neuronal specific markers microtubule-associated protein-2 (MAP2, dendritic marker, Millipore). At 7 to 10 *div*, neurons were transfected with tau plasmids and mito-DsRed2 2:1 using NeuroFECT^TM^ according to the manufacturer's protocol.

### Human tissue samples

Post-mortem brain samples were dissected from frozen brains of 7 AD cases (age 72.3±11.7 years, means ±s.d.) and 5 nondemented controls (ages 79.3±11.0 years) from the Emory Alzheimer's Disease Research Center. The study was approved by the Biospecimen Committee. AD was diagnosed according to the criteria of the Consortium to Establish a Registry for AD and the National Institute on Aging. Diagnoses were confirmed by the presence of amyloid plaques and neurofibrillary tangles in formalin-fixed tissue. Informed consent was obtained from the subjects.

### The mitochondrial membrane potential assay

The mitochondrial membrane potential (Δψm) was assayed by JC-1 following the previous procedure [53]. Briefly, the cells were grown on a 96-well plate to ~70% density, and then incubated for 20 min with 5 mg/ml JC-1 in culture mediums. The red and green fluorescence were detected with the BioTek multifunctional microplater (Ascent Fluoroscan, Tecan, Durham, NC). The ratio of red (excitation 550 nm, emission 600 nm) to green fluorescence (excitation 485 nm, emission 535 nm) (FL_2_/FL_1_) was calculated in triplicates.

HEK293 cells were incubated with low concentrations (20 nM) of TMRM+ in experimental medium for 30 min. At equilibrium, the fluorescence produced by this low concentration of TMRM+ (excitation 543/emission red photomultiplier channel) is a direct function of Δψm, and complications attributable to self-quenching of the dye are eliminated [54, 55]. Dye intensities in images were quantified by measuring the raw pixel intensities in cells somas with the region tool of MetaMorph software. The dye intensity measured in each cell was normalized to the average dye intensity of control cells receiving the same concentration of dye for the same period as the experimental cells. Normalized data are shown as fold change from the average intensity of the dye measured in control cultures at the same time.

### Preparation of mitochondria

Mitochondria were isolated as described [56]. Briefly, HEK293 cells were washed two times with Phosphate-Buffered Saline (PBS), and resuspended in ice cold permeabilization buffer containing 200 mM mannitol, 70 mM sucrose, 1 mM EGTA, 10 mM Hepes, 1 mM PMSF (Sigma) and protease inhibitor cocktail (Sigma). Cells were homogenized on ice with a 1 ml Insulin syringe 27G1/2, drawing through the needle 20 times. Following centrifugation at 600 g at 4°C for 10 min, the supernatant was collected and centrifuged at 8,000 g at 4°C for 15 min. The supernatant containing cytoplasmic proteins was collected in 12.5% trichloroacetic acid (Sigma) and stored at −20°C. The pellet (mitochondrial fraction) was washed two times with permeabilization buffer and the mitochondria were lysed with 1×cell lysis buffer (50 mM Tris-HCl (pH 7.4), 150 mM NaCl, 5 mM EDTA, 0.1% Triton X-100), plus 1 mM PMSF and protease inhibitor cocktail.

### The mitochondrial topology assay of tau

To examine the topology of tau in mitochondria, the isolated mitochondria were resuspended in sodium carbonate (pH 11.5) or the lysis buffer and incubated on ice for 30 min, and then centrifuged for 5 min at 20,000×g, the supernatant were collected in 12.5% trichloroacetic acid. For trypsin digestion, the isolated mitochondria (60 μg) were incubated with trypsin (60 μg/ml) for 30 min on ice in a final volume of 200 μl in the presence or absence of 1% triton X-100. The reaction was stopped by addition of 10 μl of trypsin inhibitor (10 mg/ml stock), or separation of the organelles from the trypsin solution by centrifugation. The resulting pellets were then resuspended in lysis buffer, briefly sonicated prior to determining the protein concentration using the BCA assay (Pierce) with bovine serum albumin as standard. Equal amount of protein (20 μg) from each reaction was analyzed by Western blotting. The antibodies against, cytochrome c oxidase (COXIV) and cyt c were used as markers of the outer mitochondrial membrane, inner membrane proteins and intermembrane space protein markers, respectively [31].

### Western blotting

Equal amounts of protein were separated by 10% sodium dodecyl sulfate-polyacrylamide gel electrophoresis (SDS-PAGE) and transferred onto nitrocellulose membranes. The membranes were blocked in 5% non-fat milk for 1 h at room temperature and then incubated with primary antibody at 4°C overnight. Then the blots were incubated with IRDye 800CW-conjugated affinity-purified anti-mouse IgG (Rockland) and IRDye 800CW anti-rabbit IgG secondary antibody (Rockland) for 1 h at room temperature. Immunoreactive bands were visualized using the Odyssey Infrared Imaging System (Licor Biosciences, Lincoln, NE, USA).

### Reverse transcription and real-time quantitative PCR

Reverse transcription and real-time quantitative PCR were carried out according to manufacturer's instruction (TaKaRa, Dalian, China). The PCR system contains 3 mM MgCl_2_, 0.5 μM forward and reverse primers, 2 μl SYBR Green PCR master mixes and 2 μl cDNA, and the standards for each gene were prepared using appropriate primers by a conventional PCR. The samples were assayed on a Rotor Gene 300 Real-time Cycler (Corbett Research, Sydney, Australia). The expression level of the interest gene was normalized by the housekeeping gene glyceraldehyde-3-phosphate dehydrogenase (GAPDH), which was not changed by the treatments. PCR primers employed in the present study are as follow:

Atp6 forward and reverse primers, 5′-TTTCCCCCTCTATTGATCCC-3′ and 5′-GTGGCCTTGGTATGTGCTTT-3′; Rpl13 forward and reverse primers, 5′-CCGGCATTCACAAGAAGGTG-3′ and 5′-CGAGCTTTCTCCTTCTTATAGACGT-3′; GAPDH forward primer 5′-GGAGCGAGATCCCTCCAAAAT-3′ and reverse primer 5′-GGCTGTTGTCATACTTCTCATGG-3′.

### Ethics statement

Investigation has been conducted in accordance with the ethical standards and according to the Declaration of Helsinki and according to national and international guidelines and has been approved by the authors' institutional review board.

### Statistical analysis

All data were collected and analyzed in a blinded manner. Data were expressed as means±SD and analyzed using SPSS 10.0 statistical software (SPSS Inc. Chicago, IL, USA). *P*-values were calculated with unpaired Student's *t*-test (two-tailed, type 2) for one-way comparisons and with ANOVA followed by post hoc test (Student's *t*-test, two-tailed, type 2) for multiple comparisons.

## SUPPLEMENTARY MATERIAL FIGURES



## Abbreviations

AD: Alzheimer's disease
htau: wild type human full-length tau protein
Aβ: β-amyloid
Mfn1: mitofusin 1
Mfn2: mitofusin 2
OPA1: optic atrophy type 1
DLP1: dynamin like protein 1
PINK1: PTEN-induced kinase 1
HEK293: human embryonic kidney 293
DMEM: Dulbecco's Modified Eagle's medium
IP: immunoprecipitation
CCCP: carbonyl cyanide m-chlorophenylhydrazone
TMRM: etramethyl rhodamine methyl ester
PGC-1α: peroxisome proliferator-activated receptor coactivator 1α
TFAM: mitochondrial transcription factor A
