# Supplementary material for: Tau accumulation impairs mitophagy via increasing mitochondrial membrane potential and reducing mitochondrial Parkin
Source: Oncotarget. 2016 Mar 2;7(14):17356–68. doi: 10.18632/oncotarget.7861 (PMC4951217; doi:10.18632/oncotarget.7861)
Supplement: Supplementary file 1 [file oncotarget-07-17356-s001.pdf]

Tau accumulation impairs mitophagy *via* increasing mitochondrial membrane potential and reducing mitochondrial Parkin

Supplementary Material

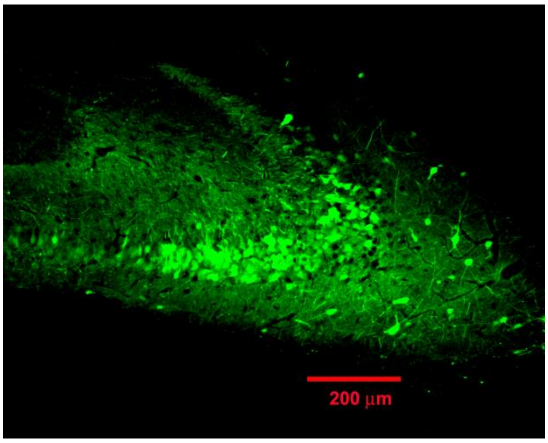

Supplementary Figure 1: The direct fluorescence image after hippocampal CA3 region infusion of eGFP labeled AAV virus for 4 weeks.

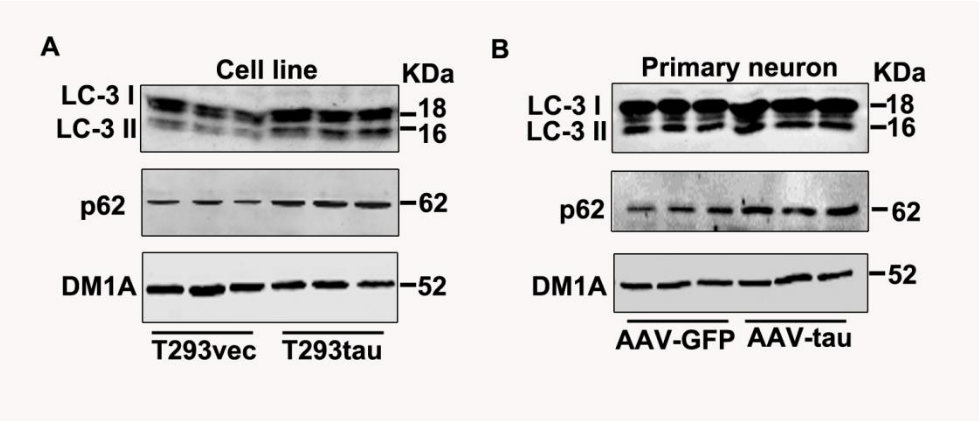

Supplementary Figure 2: Overexpression of htau increases autophagy marker protein level.

(A) The human wild type full-length tau (htau) plasmid was transiently expressed in HEK293 cells for 48 h (T293tau), (B) EGFP-labeled AAV-htau was overexpressed in primary hippocampus neurons (7 *div*) for 48 h, and then, levels of autophagy marker proteins LC-3 and p62 in the cell lysates were detected by Western blotting.

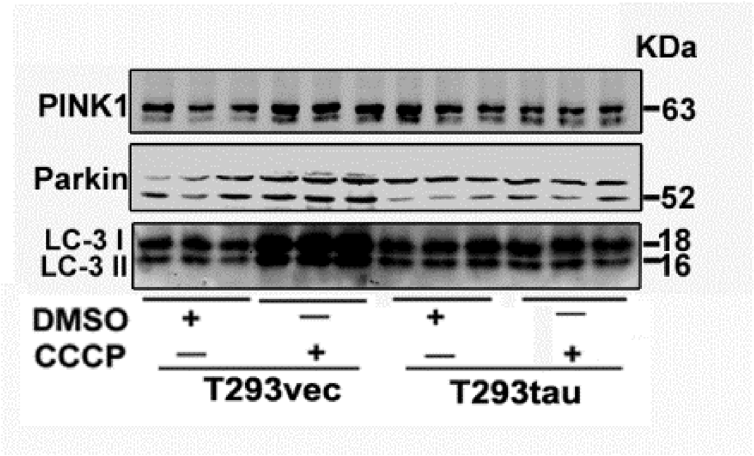

Supplementary Figure 3: CCCP treatment increased LC-3 level in T293vec cells.

Levels of PINK1, Parkin and LC-3 were detected by Western blotting in T293tau and T293vec cells after treatment with CCCP (20 μM for 30 min).

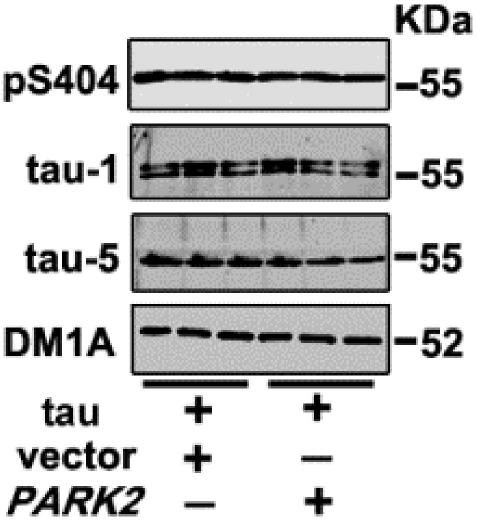

Supplementary Figure 4: Up-regulating parkin had no effect in the level of tau.

T293tau cells were transfected with wild type *parkin* (PARK2) plasmid for 48 h, and then the levels of total tau (tau-5), non-phosphorylated tau at Ser198/202 (tau-1), and phosphorylated tau at Ser404 (pS404) were measured by Western blotting.
